# The temperature arrested intermediate of virus-cell fusion is a functional step in HIV infection

**DOI:** 10.1186/1743-422X-3-36

**Published:** 2006-05-25

**Authors:** Hamani I Henderson, Thomas J Hope

**Affiliations:** 1 University of Illinois @ Chicago, Department of Microbiology and Immunology, Chicago, IL 60612, USA; 2 Northwestern University Department of Cell and Molecular Biology, Chicago, IL 60611, USA

## Abstract

HIV entry occurs via membrane-mediated fusion of virus and target cells. Interactions between gp120 and cellular co-receptors lead to both the formation of fusion pores and release of the HIV genome into target cells. Studies using cell-cell fusion assays have demonstrated that a temperature-arrested state (TAS) can generate a stable intermediate in fusion related events. Other studies with MLV pseudotyped with HIV envelope also found that a temperature sensitive intermediate could be generated as revealed by the loss of a fluorescently labeled membrane. However, such an intermediate has never been analyzed in the context of virus infection. Therefore, we used virus-cell infection with replication competent HIV to gain insights into virus-cell fusion. We find that the TAS is an intermediate in the process culminating in the HIV infection of a target cell. In the virion-cell TAS, CD4 has been engaged, the heptad repeats of gp41 are exposed and the complex is kinetically predisposed to interact with coreceptor to complete the fusion event leading to infection.

## Introduction

The fusion process of HIV envelope (Env) is a highly concerted and cooperative process between viral particles and human target cells. HIV Env mediated fusion is initiated through gp120 interactions with cell surface CD4 [1]. These interactions lead to conformational changes in Env, which expose binding sites to the principle cellular coreceptors CCR5 or CXCR4 [2]. CD4 binding also induces conformational changes in the gp41 subunit of Env, leading to exposure of the N-terminal hydrophobic fusion peptide and the heptad repeats [1]. The fusion peptide then inserts into the host cell plasma membrane, which brings the two membranes together to allow fusion. Recently, much attention has focused on events related to the fusion of viral and target cell membranes. These studies have provided insight into intermediate stages within the fusion process, which has led to the development of successful alternative drug therapies. For example, enfuvirtide (T-20) was recently approved for clinical treatment of HIV-1. T-20 is a peptide fusion inhibitor, which disrupts fusion by interacting with the N-terminal helical regions within gp41 to prevent six-helix bundle formation. Although enfuvirtide and other entry inhibitors utilize unique mechanisms to disrupt HIV entry, the virus can readily develop resistance to these compounds. Therefore, much remains to be elucidated regarding the kinetics and rate-limiting steps involved in viral fusion.

Much of the analysis of HIV fusion has been in the context of cell-cell based fusion assays. Typically, effector cells that express fusion proteins on their surface are coincubated with target cells expressing the appropriate receptor and coreceptors. Fusion between effector and target cells is measured by lipid or cytoplasmic content mixing [3]. Although these assays provide valuable information regarding fusion, it is important to fully assess all the variables governing fusion of virions to their cellular targets because of differences between virion and cellular membranes.

Research has shown that the lipid composition and fluidity of the HIV envelope membrane is significantly different from that of the host cell plasma membrane [4]. The HIV envelope membrane has an unusually high content of cholesterol and phospholipids [4]. Other findings conclude that HIV preferentially selects lipid rich domains within the host cell plasma membrane for budding from and entry into host cells [5-7]. A number of studies also support the notion that the specificity of the viral envelope membrane plays a critical role in both entry and infection by HIV virions [5,7]. Due to differences between the HIV envelope membrane as well as the plasma membrane of target cells, cell-cell fusion assays may not accurately reflect what happens during virion-cell fusion. Recently, Melikyan and colleagues were able to develop a pseudoviral-cell fusion system using time-resolved imaging of HIV-1 to monitor fusion of an individual virion to a cell [3]. This assay was based on the observed loss of a fluorescent marker located in the virion membrane. When the virion and cell membrane merge, the viral membrane label is free to diffuse in the cell membrane. In this assay, fusion is scored by a loss of membrane. This approach can provide important insights into HIV entry. However, other studies reveal that lipid mixing can take place without the completion of the fusion process. For example, for the entry of rous sarcoma virus (RSV), lipid mixing is pH-independent, while the completion of the fusion process is pH-dependent [8]. Further, the formation of a fusion pore appears to be reversible [9]. Again lipid mixing can take place without the completion of the fusion process.

Considering the potential confounding aspects of lipid mixing assays and differences between virion-cell fusion and cell-cell fusion we explored the early events in HIV entry using viral infection as the readout of successful completion of the entry process. For this analysis we took advantage of the temperature arrested state which has been previously demonstrated to represent an intermediate step in the process of fusion mediated by the interaction of HIV envelope and cells expressing CD4 and coreceptor. These studies by Melikyan et. al. demonstrated that a temperature arrested state (TAS) can be created by pre-incubating effector cells expressing HIV envelope and target cells expressing CD4 and coreceptor at suboptimal temperatures before shifting to 37°C, which is permissive for fusion [9-11]. These studies revealed a rapid increase in the fusion kinetics of effector and target cells that were initially maintained at suboptimal temperatures, compared to those cells maintained at the biologically relevant temperature of 37°C [9,11]. They found that during cell-cell fusion TAS, CD4 had been engaged and the heptad repeats in gp41 had been exposed, but coreceptor had not yet been engaged. Our analysis reveals that an analogous "temperature arrested state" can be generated for virion-cell fusion and that it is an intermediate in the process leading to HIV infection.

## Results

**Figure 1:**
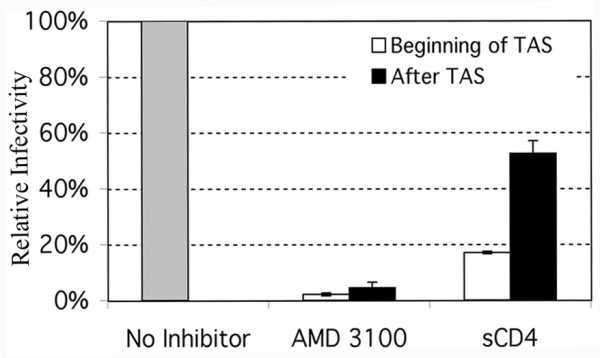
Fusion inhibition by PRO 542 and AMD 3100. Magi +/+ cells were preincubated for 2 hours at 4°C. Unbound virus was washed away with PBS. Fusion inhibitors AMD 3100 (a CXCR4 antagonist) or PRO 542 (a neutralizing antibody against gp120) were added to cells along with wild type HIV-1_NL4.3 _at the onset of the 23°C incubation period (white bars) or after 23°C-TAS was established by two hours (black bars). Inhibitors were allowed 1 hour for binding then washed. Following the 3 hour TAS incubation period, the cells were washed with PBS and shifted to 37°C to promote fusion. For control experiments (grey bar), TAS was established, subsequently shifted to 37°C, however no inhibitor was added to block fusion. The averages of triplicate experiments are shown (*n *= 4). Error bars represent the standard error of the mean. The concentrations of inhibitors were: 4 uM of AMD 3100 and 5 ug/mL of PRO 542.

To determine if TAS was an intermediate step in the process of HIV fusion leading to infection we compared the ability of known inhibitors of the interactions required for HIV envelope mediated fusion of virions bound to cells at 4°C followed by incubation at 23°C. For this analysis we used Magi +/+ cells. These cells stably express CD4 and CCR5 as well as endogenous levels of CXCR4. These cells also contain an expression cassette for β-galactosidase driven by an HIV LTR promoter. If the cells become infected, the integrated provirus expresses the HIV protein tat, which in turn activates β-galactosidase expression. Expression can then be readily detected in a liquid assay for β-galactosidase and read in a plate reader. Analysis was done in the context of a 96 well plate and the cells were infected with a 2-fold dilution series of HIV to demonstrate that the Magi +/+ assay was in the linear phase of infection and enzymatic detection (data not shown). To differentiate which steps of virus-cell fusion occurred before or after the extended 23°C (TAS) incubation, inhibition by soluble CD4 (sCD4) and the CXCR4 antagonist (AMD 3100) were utilized. Initially, target cells were incubated with HIV-1_NL4.3 _for 2 hours at 4°C to allow binding and attachment of viral particles. The unbound virus was washed away with PBS and the cells where then maintained at 23°C to establish TAS. Soluble CD4 (PRO 542), which binds to gp120 and competes with the binding of cell associated CD4, or AMD 3100 which inhibits CXCR4 binding, were added to a subset of cells during the first hour ("before TAS") at 23°C-TAS or during the last hour ("after TAS"). Inhibitors were only present for 1 hour to interact and then washed away. After the three hours at 23°C-TAS the cells were shifted to 37°C to promote full fusion. 48 hours later, infection was measured by β-gal activity. We added the inhibitors at the onset of 23°C-TAS to provide ample opportunity to alter virus-cell fusion early during the fusion process. Conversely, inhibitors were added in the last hour of 23°C-TAS to examine whether TAS allowed fusion components to proceed towards a fusion intermediate in which the inhibitors were no longer effective (figure 1). As shown in figure 1, AMD 3100 was capable of inhibiting virus-cell fusion both before and after 23°C-TAS. In contrast, sCD4 was able to inhibit infection to a greater extent when present at the beginning of the 23°C-TAS incubation period, than if present during the last hour of 23°C-TAS (figure 1). This result indicates that during the 23°C-TAS incubation, CD4 has been engaged, but interaction between HIV envelope and CXCR4 had not yet reached an irreversible state. This analysis also reveals that the much of the initial binding events of HIV to the target cells at 4°C were CD4-independent.

**Figure 2:**
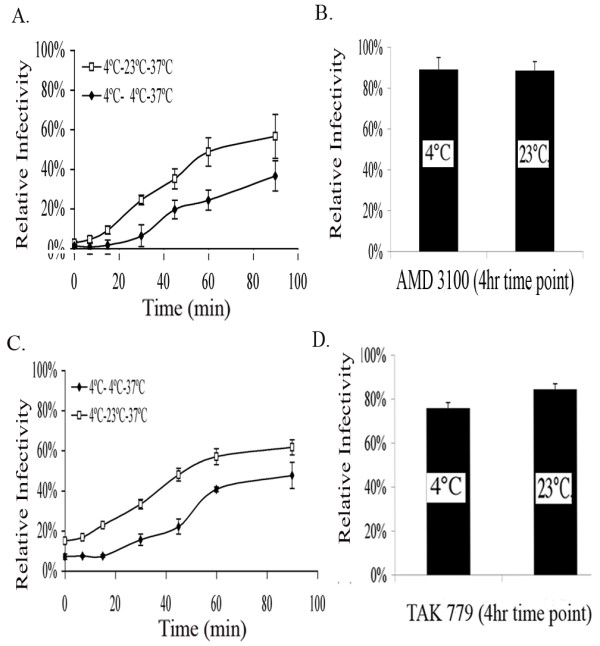
Kinetics of CXCR4 and CCR5 tropic Virus-Cell Fusion. (A) Magi +/+ cells were plated at 10,000 cells/well in two separate 96 well plates. Cells were infected with wild type HIV-1_NL4.3 _or HIV-1_JRFL _and incubated at 4°C for 2 hours to allow viral binding. Unbound virus was washed away with PBS. Both plates were incubated for an additional 2 hours; one plate remaining at 4°C, while the other plate was shifted to 23°C-TAS. Following the second 2-hour incubation period, both plates were shifted directly to 37°C to promote viral fusion. AMD 3100 (4 uM) or TAK 779 (5 ug/mL) were added at various time points to inhibit subsequent fusion. (A, C) Virus-cell fusion kinetics are faster when pre-incubated at 23°C-TAS (diamonds) compared to when cells are directly shifted to 37°C following the 4°C incubation period (squares). (B, D) CXCR4 and CCR5 fusion kinetics after 4 hours. Varying temperatures were maintained using Eppendorf Centrifuge 5810 R. Relative infectivity was quantified using a liquid β-Gal assay. OD 405 nm, optical density at 405 nm. The averages of triplicate experiments are shown (*n *= 4). Error bars represent the standard error of the mean.

Next we analyzed the kinetics of coreceptor engagement as revealed by infection becoming resistant to fusion inhibitors that disrupt interactions between HIV envelope and chemokine receptors. For these experiments, two separate 96 well plates of Magi +/+ cells were incubated with virus at 4°C for 2 hours to allow virus attachment. The virus was removed by washing with PBS. Following binding at 4°C, one 96 well plate was held at 4°C while the other 96 well plate was shifted to 23°C-TAS. Following an additional 2 hours at these temperatures both plates were shifted to 37°C to promote full viral fusion. Coreceptor antagonists were then added at various time points following the shift to 37°C. 48 hours later infection was measured by β-gal activity. The kinetics of both CXCR4 tropic and CCR5 tropic virus infection were analyzed for virus-cells preincubated at 23°C-TAS compared to those preincubated at 4°C (figure 2). For both CXCR4 tropic and CCR5 tropic virus we found that infection became resistant to drugs that target coreceptor engagement faster when cells were preincubated at 23°C-TAS. The drugs were present until the assay of β-gal activity in these experiments. Resistance to the inhibitors AMD3100 and TAK779 was observed within 7 minutes of incubation at 37°C. Conversely, resistance to the inhibitors took 30 minutes when cells were preincubated at 4°C and shifted directly to the fusion permissive temperature of 37°C (figure 2A, 2C). We also found that by adding inhibitors 4 hours after shifting to 37°C, there was no difference in the fusion kinetics of cells preincubated at 23°C-TAS compared to those preincubated at 4°C (figure 2B, D). This reveals that the cells preincubated at 4°C will eventually catch up to the cells preincubated at 23°C-TAS. These results demonstrate that TAS provides a kinetic predisposition for engagement of coreceptor by virion associated gp120.

**Figure 3:**
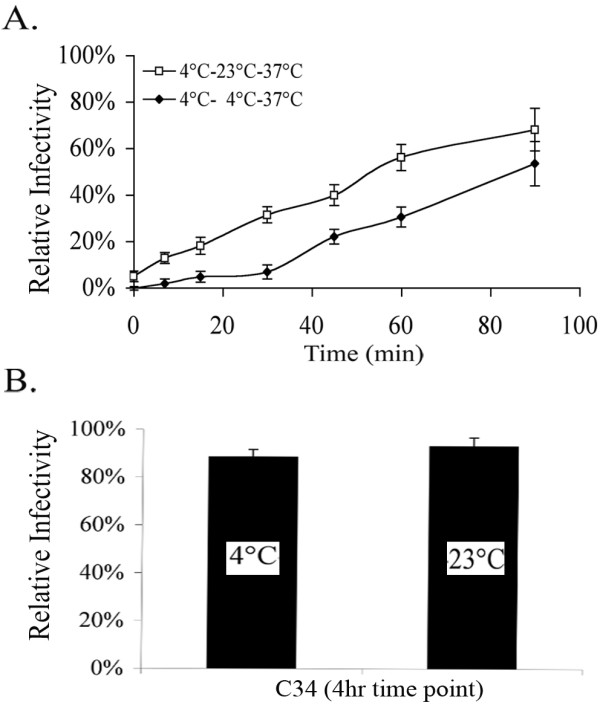
Inhibition by C34 peptide. (A) Fusion kinetics of CXCR4 tropic virus-cell fusion using the C34 peptide for inhibition. Virus-cell fusion kinetics are faster when pre-incubated at 23°C-TAS (squares) compared to when cells are directly shifted to 37°C following the 4°C incubation period (diamonds). (B) Fusion kinetics after 4 hours.

To further define the virion-cell TAS intermediate, we carried out similar experiments using the C34 peptide to inhibit fusion. C34 is a C-helix peptide, which binds to transiently exposed heptad repeats within gp41 [12-14]. Previous research has shown C34 to potently inhibit six-helix bundle formation and subsequent fusion [12-14]. Here we find that C34 is able to block fusion after coincubation of virus with target cells at TAS (figure 3, time zero). Also, consistent with figure 2, the kinetics to develop resistance to C34 inhibition were faster when virus-cell complexes were maintained at 23°C-TAS before shifting them to 37°C (figure 3a). This data suggests that TAS allows virus-cell fusion to proceed to a point that does not include six-helix bundle formation.

Exposure of the heptad repeats of gp41 is required for susceptibility to C34. We therefore wanted to determine when fusion became resistant to C34 during the 23°C incubation [9]. We therefore conducted the following experiment where virus was allowed to bind cells at 4°C for 2 hours in the absence of drug. Virus was removed by washing with PBS and the cells where shifted to 23°C for 3 hours to establish TAS. In a subset of cells, we examined the ability of C34 or sCD4 to inhibit viral fusion when added in the first hour of 23°C-TAS ("before TAS") or in the last hour of 23°C-TAS ("after TAS). In either case the inhibitor was allowed 1 hour for binding and removed by washing. After 3 hours at 23°C-TAS, cells where shifted to 37°C to promote full fusion. 48 hours later infection was measured by β-gal activity. When C34 was added at the onset of TAS ("before TAS"), a minimal 20% reduction in fusion was observed, suggesting the heptad repeats of gp41 were not fully accessible during this time. However, a greater degree of inhibition was observed when C34 was added in the last hour ("after TAS") of 23°C-TAS, suggesting that the heptad repeats do eventually become accessible to the C34 peptide during TAS. Conversely, inhibition by sCD4 was only achieved when sCD4 was added at the onset of TAS and not in the last hour of TAS. This indicates that the step of sCD4 inhibition arises before TAS while the step of C34 inhibition arises after TAS has been established. Further, the lack of inhibition by C34 when added before 23°C-TAS implies that binding sites within gp41, to which C34 is reactive, are not exposed until after TAS has been established.

## Discussion

**Figure 4:**
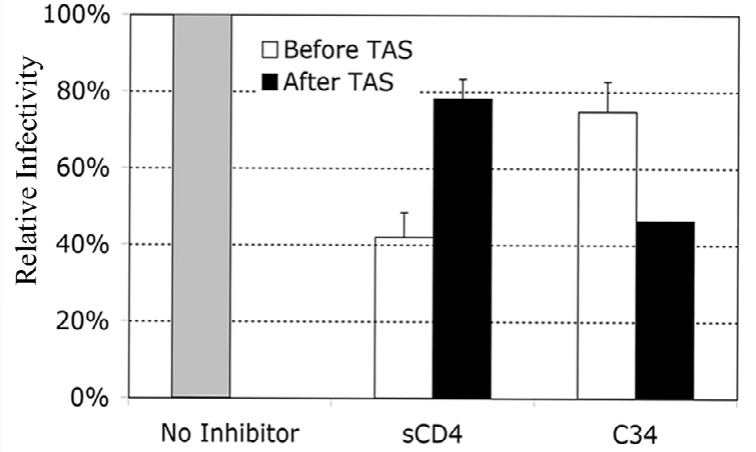
TAS contributes to the exposure of binding sites within gp41 necessary for inhibition by C34 peptide. MAGI +/+ cells were plated at 10,000 cells/well, and coincubated with HIV-1_NL4.3 _at 4°C for 2 hours. Unbound virus was washed with PBS, and cells were shifted to 23°C-TAS for 3 hours. Fusion inhibitors sCD4 (PRO 542) and C34 peptide were either added at the onset of 23°C incubation (white bars) or after TAS had been established for 2 hours (black bars). Inhibitors were allowed 1 hour for binding, then washed. Following the 3 hour TAS incubation, the cells were washed with PBS and shifted to 37°C. The averages of triplicate experiments are shown (*n *= 4). Error bars represent the standard error of the mean. Relative infectivity was quantified using a liquid β-Gal assay. OD 405 nm, optical density at 405 nm. The concentrations of the inhibitors were: sCD4 (5 ug/mL) and C34 (100 nM).

The fusion of the HIV membrane with that of a target cell is a complicated multistep process requiring a variety of molecular interactions. Previous studies show that a temperature sensitive intermediate can be generated for the HIV fusion process following prolonged incubation at temperatures ranging from 18°C to 23°C using cell-cell based fusion assays. In the TAS identified by Cohen and coworkers, CD4 has been engaged to a lesser extent than coreceptor. This is revealed by sensitivity to compounds that block interactions between the HIV envelope and receptor/coreceptor [9,15]. Likewise, the heptad repeats are exposed after CD4 engagement by HIV envelope [16]. The ability to generate a similar intermediate using MLV pseudotyped with HIV envelope has recently been reported [17]. However, in this case, the HIV envelope lacked its normal c-terminal tail, which was removed to allow incorporation into MLV based particles. The infectivity of these pseudotyped virions was not analyzed in this study. In the studies presented here we find that a TAS can be generated in the context of wild-type HIV infection of a target cell. Similar to the cell-cell based TAS, the extended incubation of HIV with target cells at 23°C led to a decrease in sensitivity to inhibition by soluble CD4. We also observed an increase in sensitivity to inhibition by the C34 peptide, which binds to the exposed heptad repeat (figure 4). Both of these changes in sensitivity were observed for the TAS generated in cell-cell fusion [9]. Additionally, like cell-cell fusion, coreceptor has not been engaged to the same extent. Since the readout for fusion in this analysis is HIV infection of target cells, it suggests that the TAS is an intermediate in the steps leading to functional virion fusion and subsequent infection.

Other studies presented here demonstrate that virions in the TAS intermediate are kinetically predisposed to pass beyond a point where they are sensitive to inhibition by CXCR4 or CCR5 coreceptor antagonists (figure 2). The same is true for the fusion passing beyond a point where it is sensitive to inhibition by the C34 peptide (figure 3). The most simple interpretation of this finding is that the time needed for envelope to successfully engage cellular receptors is eliminated during the 23°C incubation. Therefore, the fusion complex can proceed directly to complete coreceptor engagement, and go on to fuse after shifting to 37°C. In contrast, virions incubated at 4°C for the same period are delayed in achieving resistance to coreceptor antagonists because they must take the time to properly engage CD4 before proceeding to engage a coreceptor. Coreceptor engagement following CD4 engagement has been demonstrated to take approximately 30 minutes [18,19]. After 4 hours, any kinetic advantage imparted by the TAS is lost as virus-cell cultures become resistant to both coreceptor antagonists or C34 peptide, regardless of whether they were incubated at 23°C or 4°C (figure 2, 3B). The kinetics of resistance to downstream fusion inhibitors also demonstrates that TAS is an intermediary in the process leading to fusion and infection. It is possible that TAS represents a non-productive but reversible intermediate. However, reversion to the functional pathway would take time, resulting in the delay of fusion of virus-cell cultures maintained at TAS relative to control cells. Therefore, the kinetic predisposition of virus-cell cultures to advance beyond sensitivity to downstream inhibitors of fusion demonstrate that the TAS intermediate represents a discreet step in the fusion pathway which ultimately leads to HIV infection of target cells.

Comparing our findings using virus-cell interactions to previous studies using cell-cell based fusion assays reveals a difference between the cell-cell TAS and virus-cell TAS at the level of engagement of the CXCR4 coreceptor. In the cell-cell based assays, there is a significant and detectable engagement of the coreceptor shown by resistance to the CXCR4 binding peptide T22 [9]. Likewise, significant resistance to AMD3100 for blocking CXCR4 mediated fusion after incubation at 23°C for 2 hours is also observed. Furthermore, Mkrtchyan has shown that the longer the incubation period, the greater the extent of resistance [15]. In contrast, we have shown that incubation of virus and target cells at 23°C after 2–3 hours confers negligible resistance to AMD3100. Conversely, our studies with CCR5 tropic envelope in the virus-cell TAS, mimicked previous findings using a TAS for cell-cell fusion assays. For example, resistance to TAK779 was 20% after incubation at 23°C (figure 2C) similar to the cell-cell fusion studies. One possible explanation for this difference lies in the mobilities of the chemokine receptors in the membrane. We have previously reported that CCR5 is highly mobile in the membrane [20]. In contrast, we have recently found that CXCR4 in much less mobile [21]. The highly mobile CCR5 can begin to engage the CD4 bound HIV envelope at 23°C while the less mobile CXCR4 can not. The mobility of CXCR4 is less important in the case of the cell-cell assays because it would be recruited to the site of cell-cell contact within minutes of the shift to 37°C, as has been reported for the virological synapse. It has previously been shown that receptor/co-receptor density plays a role in the rate of HIV fusion and infection [22], and that multiple receptor and co-receptor molecules must engage multiple gp120 subunits in order to initiate fusion [23]. The formation of a virological synapse recruits CXCR4 and increases the rate of these engagements. The difference observed in virus-cell interactions suggests that CXCR4 is not actively recruited to the site of virus binding at the same rate.

Studies by Kabat's laboratory suggest that CCR5 entry is governed by three kinetic processes. One of these processes includes the formation of 'competent complexes.' These 'competent complexes' consist of sufficient CCR5-gp120 associations that are capable of proceeding further into the fusion process. The TAS intermediate reveals that the interaction with chemokine receptor is the rate-limiting step in the process of the formation of these 'competent complexes'. Potential temperature sensitive steps might actually be differences in chemokine mobility or affinity to the HIV envelope. It is unlikely that the temperature sensitive step is associated with conformational changes known to take place in HIV envelope because of the differences observed between cell-cell TAS and virus-cell TAS described above. The TAS intermediate described here allows the HIV-1 fusion reaction to be analyzed in the context of infectious HIV-1 particles and their respective target cells. Using suboptimal temperatures, we can gain valuable insight into HIV-1 virus-cell fusion kinetics. Ultimately, generating a temperature-arrested intermediate for virus-cell fusion provides a useful tool for synchronizing entry and studying HIV-1 fusion microscopically.

## Materials and methods

### Virus-cell fusion/infection assays

In order to study virus-cell fusion, we developed a system that allows fusion to be assayed in the context of infectious virions that were preincubated with target cells. The Magi reporter cell line for viral infection was employed [24]. Magi +/+ cells derive from HeLa cells and stably express CD4 and CXCR4 on the cells surface. Magi +/+ cells also contain a stably integrated copy of the β-galactosidase (β-gal) gene downstream of the HIV-1 long terminal repeat (LTR). Upon infection, the HIV transactivator protein Tat activates β-gal expression. Therefore, viral fusion leads to β-gal expression and the level of β-gal activity can be measured from infected cells [24]. 36 hours post infection, cells were lysed in sodium phosphate buffer with 0.2% Triton X and assayed for β-gal expression. β-Gal expression was quantified by monitoring cleavage of the colorimetric β-gal substrate *o*-nitrophenyl-β-D-galactopyranoside (ONPG) using a 96-well microplate reader measuring absorbance at 405 nm. The average background values of uninfected cells were subtracted from the values of infected cells. Varying temperatures were maintained using the Eppendorf Centrifuge 5810 R. Using this system, we manipulated temperature conditions during virus-cell incubation and used fusion inhibitors to explore kinetic factors influencing HIV-1 entry.

### Cell culture and virus production

Magi +/+ cells were grown in Dulbecco's modified Eagle's growth medium (BioWhittaker, Walerville, Md.), which contained 10% fetal bovine serum and 1% penicillin-streptomycin-glutamine. Virus was produced by CaPO_4 _transfection of 293T cells with 20 μg of HIV-1_Bru _or HIV-1_JRFL _proviral constructs. Two days following transfection, virus was harvested through a 0.45 μm pore sized filter.
